# Differential Preparation Intervals Modulate Repetition Processes in Task Switching: An ERP Study

**DOI:** 10.3389/fnhum.2016.00057

**Published:** 2016-02-18

**Authors:** Min Wang, Ping Yang, Qian-Jing Zhao, Meng Wang, Zhenlan Jin, Ling Li

**Affiliations:** Key Laboratory for NeuroInformation of Ministry of Education, School of Life Science and Technology, University of Electronic Science and Technology of ChinaChengdu, China

**Keywords:** repetition suppression, repetition priming, task-switching, P3, response-stimulus interval (RSI), cue-stimulus interval (CSI)

## Abstract

In task-switching paradigms, reaction times (RTs) switch cost (SC) and the neural correlates underlying the SC are affected by different preparation intervals. However, little is known about the effect of the preparation interval on the repetition processes in task-switching. To examine this effect we utilized a cued task-switching paradigm with long sequences of repeated trials. Response-stimulus intervals (RSI) and cue-stimulus intervals (CSI) were manipulated in short and long conditions. Electroencephalography (EEG) and behavioral data were recorded. We found that with increasing repetitions, RTs were faster in the short CSI conditions, while P3 amplitudes decreased in the LS (long RSI and short CSI) conditions. Positive correlations between RT benefit and P3 activation decrease (repeat 1 − repeat 5), and between the slope of the RT and P3 regression lines were observed only in the LS condition. Our findings suggest that differential preparation intervals modulate repetition processes in task switching.

## Introduction

Repetition suppression is associated with a reduction of neuronal firing rates when stimulus characteristics are repeated (Henson, 2003; Grill-Spector et al., 2006; Krekelberg et al., 2006). Studies have shown that repetition suppression is closely related to neuronal adaptation in prefrontal and parietal areas (Rainer and Miller, 2000; Vuilleumier et al., 2002; Lehky and Sereno, 2007; De Baene et al., 2012), and is generally thought to be associated with neural processing of behavioral facilitation effects (or behavioral priming effects; Henson, 2003). However, increasing number of studies have challenged the conventional interpretation that repetition suppression only reflects neuronal adaptation, a relatively automatic consequence of the bottom-up flow of perceptual information processing (Wiggs and Martin, 1998; Grill-Spector et al., 2006). Instead it has been proposed that a diverse range of cognitive factors might be also associated with the neuronal population response changes in repetition suppression, such as attention, stimulus recognition, learning, expectation and explicit memory (Nakamura et al., 2007; Buchsbaum and D’Esposito, 2009; Davis et al., 2009; Larsson and Smith, 2012).

Besides the repetition effect observed in concrete stimulus presentation (i.e., consecutive repetition of shape images; Wagner et al., 2000; Huettel et al., 2002; Summerfield et al., 2008), a recent fMRI study used a parametric approach to show that an adaptation process to abstract rule representations may exist in repeated trials of task-switching (De Baene et al., 2012). In this study, instead of successively shifting between conflicting stimulus-response (S-R) mappings, the authors used longer sequences of trials for both switch and repeat conditions, in order to dissociate switch-specific differences in brain activation that were related to either adaptation or reconfiguration. However, how the rule representation can be keep in mind and facilitate the behavior is unclear.

Our current study examined the repetition priming/repetition suppression phenomena of abstract rule representations in the context of task-switching, using a previously reported paradigm (Li et al., 2012). This former study used a dual cued task-switching paradigm with long sequences of repeated trials to explore the neural mechanisms underlying the cost of task switching (Li et al., 2012). The same type of stimulus characters were successively presented at least 6 times and up to 12 times before switching to another task type. This novel design made it possible to examine the repetition processes in long sequences of repeated trials of task-switching.

Many electrophysiological task-switching studies have focused on the influence of different time sets on switch cost (SC), and on the ERP components involved in the processes associated with anticipatory and post-stimulus task-set reconfiguration (Karayanidis et al., 2003; Nicholson et al., 2005, 2006a). The duration of the response-stimulus interval (RSI) is thought to reflect processes associated with passive dissipation of the previously relevant task set (Nicholson et al., 2005; Li et al., 2012). On the other hand, the duration of the cue-stimulus interval (CSI) modulates the processes involved in task-set reconfiguration (a set of processes involved in shifting from a readiness to perform task A to a readiness to perform task B; Karayanidis et al., 2003; Rushworth et al., 2005; Li et al., 2012). However, the effects of different preparation intervals on the repetition processes in task-switching, remain largely unknown.

To examine whether different preparation intervals modulate repetition processes in task switching, the RSI and the CSI were manipulated in short and long duration conditions independently (RSI: 750 ms, 1200 ms; CSI: 150 ms, 600 ms; Meiran, 2000; Karayanidis et al., 2003; Nicholson et al., 2005). We manipulated RSI and CSI to detangle the proactive interference and stimulus preparation, two factors which may contribute to repetition effect in task-switching. In our previous study, short CSI resulted in a larger SC of reaction times (RTs; RT for the switch trial − RT for the repeat trial) compared to long CSI (Li et al., 2012), due to an inadequate preparation for the subsequent task (Nicholson et al., 2005). In other words, the duration of the CSI may affect the preparation process for the subsequent task in sequential trials (Meiran, 2000). Therefore, we speculated that short CSI will result in longer RTs compared with long CSI in repetition sequences, and that practice repetitions may improve behavioral performance, as reflected by decreased RTs with increasing number of repetitions. Furthermore, we expected that in the long CSI condition, there would be no repetition priming since the behavioral performance will have reached those of the short CSI after several repetitions due to adequate preparation. In addition, in our previous study, RT SC showed no difference between short (750 ms) and long (1200 ms) RSIs (Li et al., 2012), suggesting that 750 ms is sufficient to obtain a behavioral response. Therefore, we speculated that RTs would not differ between the short and the long RSI conditions in the repetition sequences.

ERP studies using cued task-switching paradigms have reported a parietal switch-related positivity (P3) emerging around 400–500 ms after cue onset (Rushworth et al., 2002, 2005; Miniussi et al., 2005; Nicholson et al., 2005; Li et al., 2012), reflecting processes involved in task set reconfiguration or abstract rule representations (Nicholson et al., 2005; Karayanidis et al., 2010). Such reconfiguration processes will normally not be necessary when the same task is repeated, since the cognitive system has been configured for the new task at hand (De Baene et al., 2012). Thus in the repetition sequences, cue-related P3 would either show a decrease trend or remain at a steady level as the number of repetitions increase. Long CSI may provide adequate time to complete the transformation process from the abstract rule representation (cue) in the instruction phase to the pragmatic representation (stimulus) in the repetition phase (Ruge and Wolfensteller, 2010), thus, there is no additional adaptation after the first repetition, resulting in no P3 amplitude change as the number of repetitions increases. On the other hand, in the short CSI condition, subjects may have inadequate preparation time to complete the transformation and additional adaption processes may be needed, thus the P3 amplitude decreases as the number of repetitions increase until a stable cognitive level is reached. In the present study, RSI was manipulated while CSI remained constant resulting in two conditions: SS (short RSI and short CSI) and LS (long RSI and short CSI). Long RSI have been shown to weaken the proactive interference (Nicholson et al., 2005; Li et al., 2012), so that the activities of the current trial are not affected by the continued activation of the previous trial (no positive priming), while in the short RSI the activation of the previous trial may continue throughout the current trial and thus the P3 amplitude does not decrease as the number of repetitions increases. We hypothesized that in the LS condition (long RSI and short CSI), cue-related P3 amplitude (300–450 ms post cue onset) will decrease as the number of repetitions increase due to fully overcoming interference from a previous trial and due to insufficient preparation time for the next trial.

## Materials and Methods

### Subjects

Fourteen right-handed students from University of Electronic Science and Technology of China (UESTC) were recruited for monetary compensation. All the subjects (males, mean age 25, range: 20–27) had normal color vision and had no history of neurological or psychiatric problems. Two subjects were excluded from ERP analyses due to a lack of trials (less than 40% trials); therefore, data of the 14 remaining subjects was used for behavioral analysis and the 12 remaining subjects were used for ERP analysis. The study was approved by the University of Electronic Science and Technology of China Ethics Board. Written informed consent was obtained from each subject prior to being tested. The methods were carried out in accordance with the approved guidelines and all experiments conformed to the Declaration of Helsinki.

### Stimuli and Task

**Figure 1:**
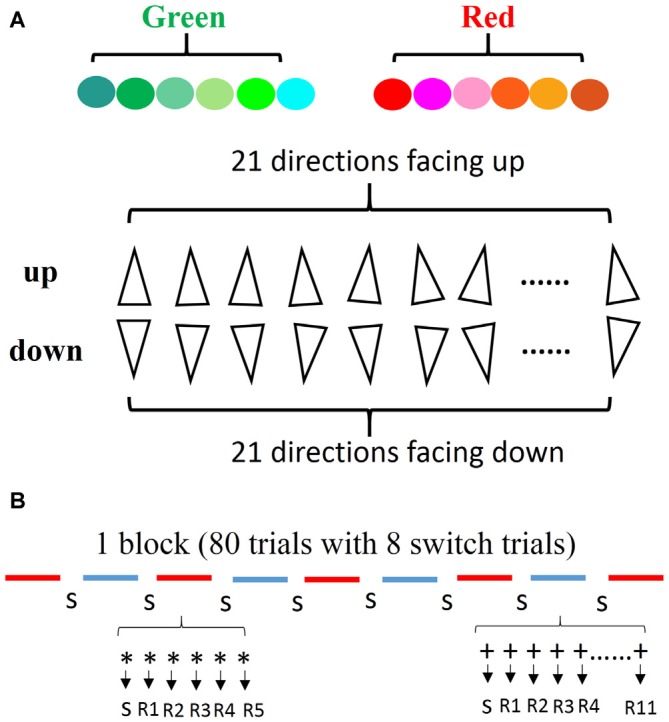
**Stimulus and the definition of repetitions. (A)** The colors and directions of stimuli used in the experiment. **(B)** The line segments indicate the type of task (e.g., red for color task, blue for direction task). The number of each type of task was counterbalanced across blocks. During each task segment, the first trial indicates a switch trial and the second is defined as the 1st repetition, thus each task type was repeated randomly from 5 to 11.

The stimuli consisted of triangles characterized by two types of color (red and green) and two types of directions (up and down). We introduced a comprehensive stimulus type to encourage subjects to use general rules rather than “check tables” or memorization strategies for solving the tasks. The color type “red” consisted of six similar colors (red, orange, pink, brown, rose, and salmon pink), and the color type “green” consisted of six similar colors (green, sea green, emerald green, aqua, turquoise, and cyan). The two types of directions included 21 directions facing up and 21 directions facing down (with a deviation of within 10 degrees). Combining the two color types with the two direction types, we obtained four types of triangles: red up (RU); red down (RD); green up (GU); and green down (GD). These stimuli were randomly selected without replacement from a set of 12 colors and each was oriented randomly in one of the 42 directions (Figure 1A).

**Figure 2:**
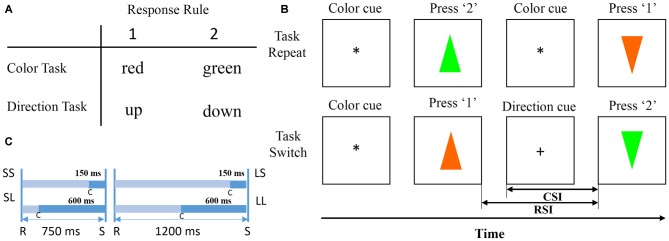
**Stimulus and trial design. (A)** Stimuli and response. Participants press the “1” key for red in the color task and for up in the direction task; the “2” key for green in the color task and for down in the direction task. **(B)** Schematic experimental paradigm (Li et al., 2012). Each trial began with a cue and ended with a response. Once a response to a previous trial was completed, the cue for a current trial appeared for a designated duration, followed by a colored and oriented triangle at the center of the screen. Participants discriminated the stimulus direction or color. **(C)** Cue-stimulus interval (CSI) and response-stimulus interval (RSI) were divided into four types: SS, SL, LS, LL. The first letter indicated whether the RSI was short (750 ms) or long (1200 ms), and the second letter indicated whether the CSI was short (150 ms) or long (600 ms).

Figure 2B illustrates an example of trial sequences. All the trial sequences were initiated by a cue (“*” or “^+^”) presented at the central of the screen indicating the task type (“*” for color task, “+” for direction task), followed by one of the equally chosen colored and oriented triangles. Subjects were required to perform the color or direction discrimination task according to the sign cue. The central cue “*” indicated a color discrimination task and “+” indicated a direction discrimination task. After a response was made, the triangle disappeared immediately, and then the cue in the next trial was presented after a designated time had elapsed. If a current cue indicated the same task as the previous one, the current trial was defined as a repeated trial. Likewise, if a current cue indicated a different task from previous one, the current trial was defined as a switch trial. Subjects were required to discriminate the color or the direction by pressing the keys “1” and “2” on a computer keyboard with the index or middle fingers of their right hand. In the color task, button 1 was used to respond to red and button 2 was used to respond to green, and in the direction task button 1 was used to respond to triangles facing up and button 2 to respond to triangles facing down (Figure 2A). RSI and CSI were defined as the interval between a previous response and a stimulus, and between a cue and a stimulus. The two levels of RSI (short, 750 ms; long, 1200 ms) and CSI (short, 150 ms; long, 600 ms) were combined to produce the following four sessions: SS, SL, LS, LL. The first letter indicated RSI length, and the second letter indicated CSI length (Figure 2C). For each block, RSI and CSI were held constant, and a Latin square design was used to avoid sequence effects. S-R mappings and cue-task associations remained constant for each subject throughout the experiment and were counterbalanced across subjects.

### Procedure

There were two practice stages. Firstly, all subjects started with two single-task blocks. Half of the participants first performed the direction task block, while the other half began with the color task block. Secondly, the subjects performed the mixed-task blocks comprising of 80 trials with eight switch trials in each block. The amount of repeated trials varied randomly from 5 to 11 (Figure 1B). Half of the subjects started with the direction task in the mixed block, while the other subjects began with the color task. In order to ensure a familiarity with the cue-task and the response-finger mapping, subjects were given enough practice to achieve a level of 90% accuracy for practice mixed blocks. After the practice sessions, four formal sessions which consisted of eight mixed-task blocks were performed by the subjects, in which 576 repeated trials and 64 switch trials were recorded in each condition. The first task in the successive blocks was alternated, for example, the first block began with the direction task, and the next block started with the color task. During the experiment, subjects were required to maintain central fixation in order to identify the cue and to respond as fast and accurately as possible. Moreover, subjects were not made aware of the RSI and the CSI before the experiment. Automatic perception and the flexibility of different time sets were required.

### Data Collection and Preprocessing

Behavioral data were recorded with E-prime 1.0 software while electroencephalography (EEG) signals were collected using a 128-channel EGI system referenced to Cz (129th) electrode. Sampling rate was 1000 Hz, and band pass was 0.1–48 Hz. An FIR 0.1–30 Hz bandpass filter was applied. Bad channels were excluded if the remaining trials were less than 50% of all the trials. Trials associated with an incorrect response and trials with a button press occurring outside a 300–1500 ms window after the onset of the stimulus were excluded. Eye movements, blinks and muscle artifact were excluded by automatic artifact rejection (greater than 100 μV). After this preprocessing, a mean of 431 ± 32, 408 ± 31, 412 ± 27 and 423 ± 36 repeated trials remained across subjects for SS, SL, LS and LL conditions, respectively (mean ± SD). Each session of the original EEG files was segmented into eight blocks, and each trial was created by extracting 1000 ms epochs around the onset of the cue (both the color cue and the direction cue; −200 to 800 ms). Baseline was set to 200 ms pre-onset of the cue. Data were re-referenced against the average of all channels. Individual averaged ERPs were acquired by selectively averaging across both tasks (color and direction) for each repetition (from 1 to 5) in each of the four conditions (SS, SL, LS and LL). Hence there were 20 repeat-related average ERP waveforms for each subject.

### Behavioral Data Analysis

Mean reaction times (RTs) and accuracy for repeated trials (from 1 to 5) in four conditions (SS, SL, LS, LL) were analyzed initially using a 4 (condition) × 5 (repetition) × 2 (task: color, direction) repeated measure analysis of variance (ANOVA). As task did not interact with any other factor, all further analyses were averaged across tasks. In order to uncover the underlying mechanisms of repetition processes in the four conditions, the RTs and accuracy data, collapsed across both color task and direction task, were analyzed with a one-way ANOVA across the number of repetitions for each condition. We examined the differences in RTs and accuracy between conditions for each of the five repetition levels separately using a one-way ANOVA with condition as factor. *Post hoc* *t*-tests with Bonferroni correction for multiple comparisons were applied when necessary. The correlation between RTs and the number of repetitions was evaluated using Pearson correlation for each of the four conditions. Mean values ± standard error of the mean (SEM) are used for the behavioral and ERP results. All statistical analysis was performed using SPSS Statistics Release 19 (IBM, Somers, NY, USA).

### ERP Analysis

Data at midline sites (FCz, Cz, Pz) and bilateral posterior sites (PO7, PO8) were analyzed for five repetition trials in each of the four conditions according to the topographical distribution of grand average ERP activity. The midline sites were of interest in the analysis of the P3 component in both the task switching and repetition studies (Nicholson et al., 2005; Guo et al., 2007; Rozenkrants et al., 2008; Jamadar et al., 2010; Jiang et al., 2013). The posterior sites were used in the analysis of N1 and P1 components in order to examine early visual processing (Mangun and Hillyard, 1991). The peak amplitudes of N1 and P1 in the 100–200 ms and 200–300 ms time-windows, respectively, were evaluated at posterior sites. For each potential a 4 (condition) × 5 (repetition) × 2 (electrodes: PO7, PO8) repeated measure ANOVA was performed. The peak amplitudes of P3 in the 300–450 ms time-window were evaluated at electrode sites FCz, Cz and Pz and were used to perform a 4 (condition) × 5 (repetition) × 3 (electrodes: FCz, Cz, Pz) repeated measure ANOVA. Further analysis of ERP data were restricted to the sites FCz, Cz and Pz, since no reliable effects were observed between experimental conditions at posterior sites. *Post hoc* *t*-tests with Bonferroni correction for multiple comparisons were applied when necessary. Pearson correlation was used to evaluate the relationship between behavioral performance and ERP activity. Specifically, the decrease of P3 amplitude at Cz (repeat 1 – repeat 5) was correlated with RT benefit (repeat 1 – repeat 5). In addition, we calculated the slopes of the function relating RTs to repetition times and the slopes of the function relating P3 at Cz to repetition times for each of the 12 subjects who have both behavioral and ERP data. Thus 12 slope values were obtained for RTs and 12 slope values were obtained for P3 amplitudes. Then Pearson correlation was used to evaluate the relationship between the RT slopes and the P3 slopes in each of the four conditions. All regression analyses used Pearson’s linear correlation coefficient.

In order to confirm that the results of Cz electrode were reliable and in order to avoid loss of statistical power, six electrodes around Cz were pooled to form a topographical region. The P3 component was measured at this central location using a one-way ANOVA with repetition as factor in each of the four conditions.

## Results

### Behavioral Results

The mean accuracy was high across subjects (up to 95.68%). No significant differences were observed across the number of repetitions in each condition nor between conditions for each of the five repetition levels (all *p* > 0.1). Comparison of RTs, showed no significant interactions between task and condition [*F*_(3,39)_ = 2.286, *p* = 0.135] and between task and repetition [*F*_(4,52)_ = 3.018, *p* = 0.276]. As task did not interact with any other factor, all further analyses were averaged across tasks. Two-way ANOVA with condition (SS, SL, LS, LL) and repetition (from 1 to 5) as factors revealed a main effect for condition [*F*_(3,39)_ = 10.427, *p* = 0.002], a main effect for repetition [*F*_(4,52)_ = 4.192, *p* = 0.030] and an interaction between condition and repetition [*F*_(12,156)_ = 2.267, *p* = 0.028]. Therefore, a one-way ANOVA (1 × 5) with repetition as factor was used in each of the four conditions. This comparison showed a main effect for the number of repetitions in the SS (short RSI, short CSI) [*F*_(4,52)_ = 5.659, *p* = 0.012] and LS (long RSI, short CSI) [*F*_(4,52)_ = 3.961, *p* = 0.007] conditions, which confirmed that the differences between RTs across repetition times were reliable in the short CSI conditions. No significant effects were observed in the long CSI conditions (LL: *F*_(4,52)_ = 0.235, *p* = 0.912; SL: *F*_(4,52)_ = 0.713, *p* = 0.601). *Post hoc* *t*-tests, using Bonferroni correction for multiple comparisons, revealed a significant difference between repetition 2 and repetition 4 [*t*_(13)_ = 4.565, *p* = 0.045] in the SS condition, and a significant difference between repetition 2 and repetition 5 [*t*_(13)_ = 3.607, *p* = 0.032] in the LS condition.

**Figure 3:**
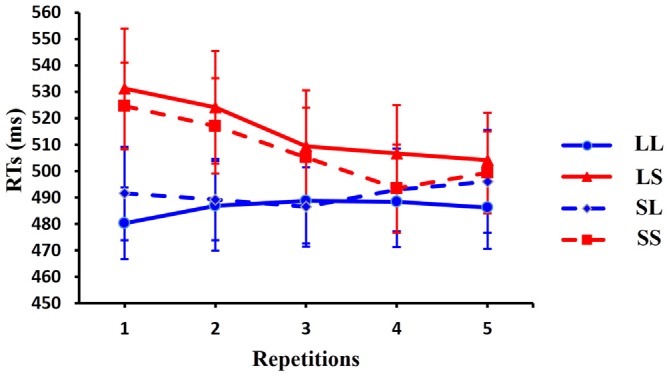
**Behavioral responses.** Average reaction times (RTs) are shown for the 14 subjects in four conditions across five repetition times. RTs are faster with increasing number of repetitions in the LS condition. Error bars show standard error of the mean (SEM).

One-way ANOVA (1 × 4) with condition as factor showed a significant main effect for repetition 1 trials [*F*_(3,42)_ = 8.635, *p* = 0.001] and for repetition 2 trials [*F*_(3,42)_ = 9.861, *p* < 0.001]. No significant effects were observed for repetition 3 trials [*F*_(3,42)_ = 3.123, *p* = 0.056], repetition 4 trials [*F*_(3,42)_ = 1.608, *p* = 0.209] or for repetition 5 trials [*F*_(3,42)_ = 1.321, *p* = 0.284]. *Post hoc* *t*-tests, using Bonferroni correction for multiple comparisons, revealed a significant difference between LL (long RSI, long CSI) and LS (long RSI, short CSI) [*t*_(13)_ = 3.608, *p* = 0.019], between LL (long RSI, long CSI) and SS (short RSI, short CSI) [*t*_(13)_ = 4.234, *p* = 0.006] and between SL (short RSI, long CSI) and SS (short RSI, short CSI) [*t*_(13)_ = 3.949, *p* = 0.010] for repetition 1 trial; a significant difference between LL (long RSI, long CSI) and LS (long RSI, short CSI) [*t*_(13)_ = 4.717, *p* = 0.002], between LL (long RSI, long CSI) and SS (short RSI, short CSI) [*t*_(13)_ = 3.637, *p* = 0.018] and between LS (long RSI, short CSI) and SL (short RSI, long CSI) [*t*_(13)_ = 3.490, *p* = 0.024] for repetition 2 trials. Furthermore, linear regression for the average RT showed that the RTs decreased linearly with the increase of repetitions in the LS condition [*R* = 0.953, *p* = 0.012] and in the SS condition [*R* = 0.916, *p* = 0.029]. Thus, RTs decreased as the number of repetitions increased in the short CSI conditions. RTs for repetition sequences across the four conditions are illustrated in Figure 3.

### ERP Results

**Figure 4:**
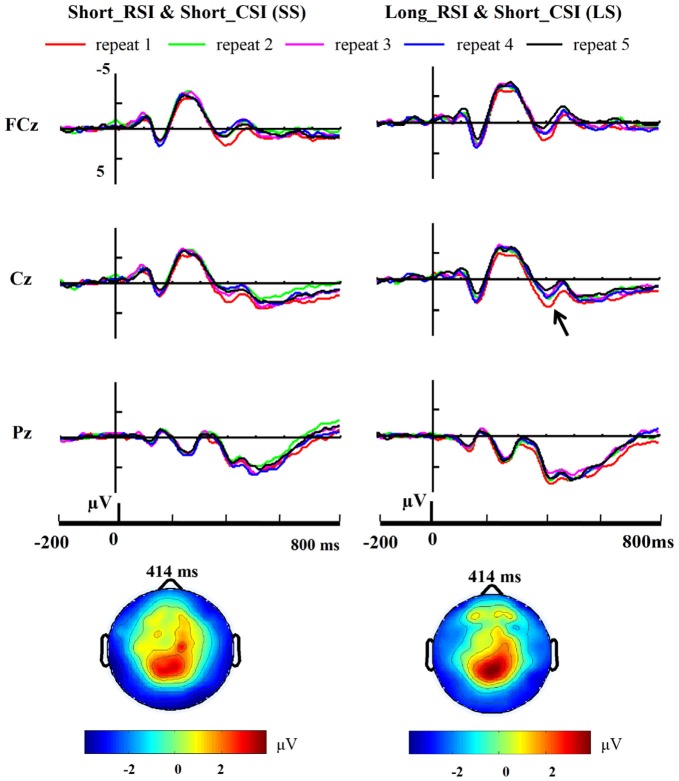
**Cue-related ERP.** Grand-average (*n* = 12) repeat-related ERP waveforms at midline electrode sites (FCz, Cz, Pz) are shown for the short CSI conditions across five repetition times. A large positivity (P3) emerged around 350 ms post cue onset at FCz and Cz.

Three-way ANOVA of P1 and N1 amplitudes with condition (SS, SL, LS, LL), repetition (from 1 to 5) and site (PO7, PO8) as factors showed no reliable effects [all *p* > 0.1]. Three-way ANOVA of P3 amplitudes with condition (SS, SL, LS, LL), repetition (from 1 to 5) and site (FCz, Cz, Pz) as factors showed an interaction between electrode and condition [*F*_(6,66)_ = 4.693, *p* = 0.041]. We then performed a 4 × 5 ANOVA with condition and repetition at each site as factors. At Cz, there was a significant main effect for condition [*F*_(3,33)_ = 9.589, *p* = 0.004], the main effect for repetition approached significance [*F*_(4,44)_ = 3.789, *p* = 0.052] and the interaction between condition and repetition also approached significance [*F*_(12,132)_ = 1.778, *p* = 0.058]. A significant main effect for condition was also observed at FCz [*F*_(3,33)_ = 28.547, *p* < 0.001] and Pz [*F*_(3,33)_ = 7.610, *p* = 0.010], but no other effects were observed for these two electrode sites. Accordingly, P3 amplitudes were evaluated and compared using a one-way ANOVA with repetition as factor in each of the four conditions at each of the sites (FCz, Cz, Pz) separately. Thus, the number of one-way ANOVAs was 12 in total. There were no significant effects for SL (short RSI, long CSI; Cz: *F*_(4,44)_ = 2.258, *p* = 0.152; FCz: *F*_(4,44)_ = 1.640, *p* = 0.255; Pz: *F*_(4,44)_ = 1.227, *p* = 0.372) and LL (long RSI, long CSI; Cz: *F*_(4,44)_ = 0.557, *p* = 0.701; FCz: *F*_(4,44)_ = 0.292, *p* = 0.875; Pz: *F*_(4,44)_ = 2.190, *p* = 0.160) conditions. There was a main effect for the number of repetitions at Cz in the LS condition [*F*_(4,44)_ = 5.058, *p* = 0.025]. *Post hoc* *t*-tests, using Bonferroni correction for multiple comparisons, revealed a significant difference between repetition 1 and repetition 5 [*t*_(11)_ = 3.708, *p* = 0.034] and repetition 2 and repetition 5 [*t*_(11)_ = 3.618, *p* = 0.041]. No main effect for repetitions was observed in the SS condition at Cz [*F*_(4,44)_ = 2.010, *p* = 0.150]. At FCz, there was a trend for a significant main effect of the number of repetitions in the LS [*F*_(4,44)_ = 3.367, *p* = 0.068] and SS conditions [*F3*_(4,44)_ = 3.557, *p* = 0.060]. *Post hoc* *t*-tests, using Bonferroni correction for multiple comparisons, revealed a significant difference between repetition 1 and repetition 5 [*t*_(11)_ = 3.956, *p* = 0.022] in the LS condition. However, no main effect of repetition was observed at Pz in both the LS [*F*_(4,44)_ = 0.573, *p* = 0.690] and SS [*F*_(4,44)_ = 1.630, *p* = 0.216] conditions. The grand-average ERPs time-locked to cue onset at midline sites (FCz, Cz, Pz) are illustrated in Figure 4 from repetition 1 to repetition 5 in the SS (short RSI, short CSI) and LS (long RSI, short CSI) conditions. One-way ANOVA of P3 amplitude with repetition as factor at central locations showed a main effect for repetition in the LS condition [*F*_(4,44)_ = 3.258, *p* = 0.020], but no significant effects were found in the other three conditions [SS: *F*_(4,44)_ = 2.409, *p* = 0.099; SL: *F*_(4,44)_ = 1.537, *p* = 0.228; LL: *F*_(4,44)_ = 0.567, *p* = 0.618]. *Post hoc* *t*-tests, using Bonferroni correction for multiple comparisons, revealed a significant difference between repetition 1 and repetition 5 [*t*_(11)_ = 3.614, *p* = 0.041] and repetition 2 and repetition 5 [*t*_(11)_ = 4.493, *p* = 0.009] in the LS condition.

**Figure 5:**
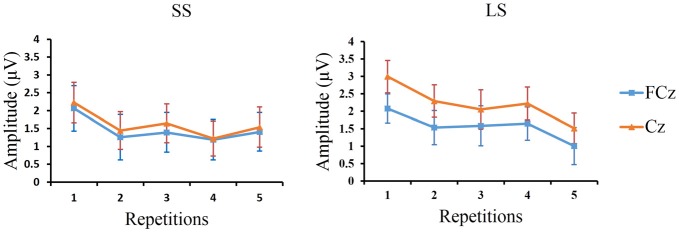
**The peak P3 amplitudes at FCz and Cz.** Grand-average peak P3 amplitudes at FCz and Cz (*n* = 12) are shown for the SS and LS conditions across the five repetition times.

Linear regression analysis was employed to reveal the variation trend of the grand-average P3 at FCz and Cz in the LS and SS conditions with increasing number of repetitions. The analysis showed that with increasing number of repetitions P3 activity decreased at Cz [*R* = 0.902, *p* = 0.036] in the LS condition but not in the SS condition [*R* = 0.674, *p* = 0.213], and only showed a tendency for this effect at FCz [*R* = 0.842, *p* = 0.073] in the LS condition (SS: *R* = 0.628, *p* = 0.257; see Figure 5).

**Figure 6:**
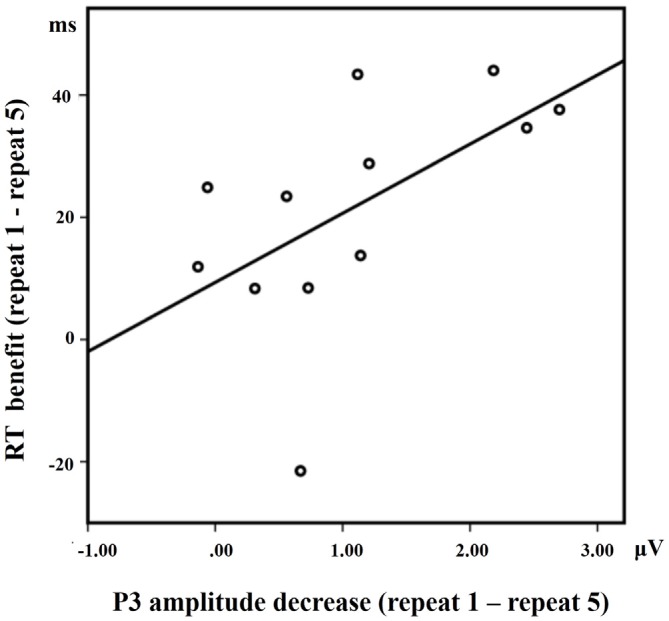
**The correlation between the RT benefit and the P3 amplitude decrease at Cz in the LS condition**.

A positive correlation between RT benefit (repeat 1 − repeat 5) and P3 activation decrease at Cz (repeat 1 − repeat 5) in the LS condition approached significance [*R* = 0.568, *p* = 0.050] for the 12 subjects who have both ERP data and behavioral data (Figure 6). Almost all the subjects showed faster RTs with the increase of repetitions, except for one subject who did not exhibit such a RT benefit. A significant positive correlation between RT benefit and P3 amplitude decreases was also observed when eliminating the data of this subject [*R* = 0.682, *p* = 0.021].

**Figure 7:**
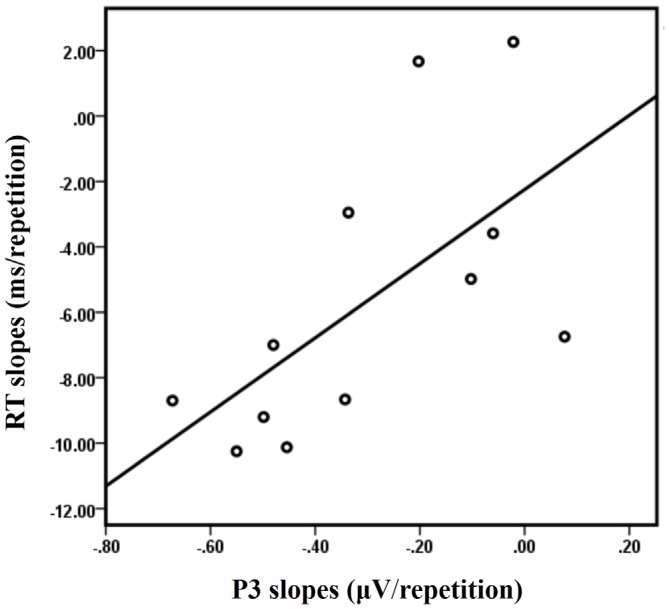
**The correlation between the RT slopes and the P3 slopes at Cz in the LS condition**.

A significant positive correlation between the RT slopes and the P3 slopes at Cz was observed in the LS condition [*R* = 0.628, *p* = 0.029] for the 12 subjects who have both ERP data and behavioral data (Figure 7). No significant correlations were observed between the RT slopes and the P3 slopes at Cz for the other three conditions (LL, SL, SS; all *R* < 0.4 and all *p* > 0.1).

## Discussion

The current study sought to identify the neuronal representations underlying repetition processes during task-switching by using ERPs. We were particularly interested in the contributions of RSI and CSI to the repetition-related changes in the long sequences of repeated trials in task-switching. We found that, as the number of repetitions increased: (a) RTs became faster in the short CSI conditions and (b) P3 amplitudes at Cz decreased in the LS condition (long RSI, short CSI). These findings suggest that repetition processes in task switching are affected by the proactive interference of the previous trial and by the readiness for the next stimulus response, which are associated with the duration of the RSI and CSI, respectively.

### Repetition Effects on Behavior

We found that RTs accelerated in the short CSI conditions, indicating a facilitated performance of repeated trials in the short CSI conditions (Sohn and Carlson, 2000; Ruthruff et al., 2001; Dreisbach et al., 2002). Furthermore, the RT differences between early repetition levels (repetition 1 and repetition 2) were only observed in the short CSI and long CSI conditions but not in the RSI conditions, suggesting that the length of CSI is the main determinant for the behavioral performance in the repeated trials. In the short CSI conditions, subjects do not have enough time to initiate the S-R mappings (Goschke, 2000; Nicholson et al., 2006b) or to complete the transformation process from the abstract rule representation (cue) in the instruction phase to the pragmatic representation (stimulus) in the repetition phase (Ruge and Wolfensteller, 2010). Thus, subjects require several repetitions to further strengthen the S-R rules and for RTs to become faster as the number of repetitions increase. However, for long preparation time intervals (CSI = 600 ms; SL, LL), RTs showed no difference between successive repeated trials, since subjects have adequate time to complete such cue-stimulus transformation processes, and thus the RT is initially already as fast as that in the short CSI after several repetitions. These findings suggest that the repetition priming effect does not always appear after repeated exposure to the same stimuli (or task), but rather depends on the extent to which the cue-stimulus transformation processes have been accomplished.

### Repetition Effects on P3

As expected, cue-related P3 amplitudes either show a decrease or remain at a steady level, as the number of repetitions increase, suggesting that the cognitive system has been configured for the new task at hand when the same task is repeated in the repetition sequences (De Baene et al., 2012). We found that cue-related P3 amplitudes decreased as the number of repetitions increased in the LS condition, replicating findings of earlier studies (Barceló et al., 2002; Rushworth et al., 2002). The reasons can be explained as follow: (1) Short CSI lack adequate time to complete the transformation process from the abstract rule representation (cue) in the instruction phase to the pragmatic representation (stimulus) in the repetition phase (Ruge and Wolfensteller, 2010) and extra adaption processes are required, thus P3 amplitudes decrease to a stable level as the number of repetitions increase. (2) Long RSI weaken the proactive interference (Nicholson et al., 2005; Li et al., 2012) so that the activities of the current trial are not be affected by the continued activation of the previous trial (no positive priming), otherwise, the neural activity of the current trial would have been a continuation of the previous repeated trial, and would not show the effects of fatigue (Grill-Spector and Malach, 2001). Thus, the modulation of P3 amplitude may reflect two sub-processes involved in the cue-stimulus transformation process of the upcoming trial and the passive dissipation of activation from the previous trial, associated with the length of CSI and RSI, respectively.

### The Correlation Between Behavior and P3 Amplitude

We found a positive correlation between the RT benefit (repeat 1 − repeat 5) and the P3 activation decrease (repeat 1 − repeat 5) at Cz in the LS condition, suggesting that the greater the decreases in P3 amplitude, the greater the RT benefit. Moreover, a positive correlation between the RT and the P3 amplitude slopes at Cz, suggests that the greater the P3 decrease the greater the reduction in reaction time in the LS condition. These results imply that cue-related P3 may be a useful index of neural adaption in long sequences of repeated trials, in which amplitude decreases are tightly correlated with RT decrease.

### Limitations and Future Directions

The current study is limited in its choice of recruiting only male subjects. Future studies are essential in order to confirm the results using both male and female subjects. In addition, more intervals are required to further dissociate between RSI and CSI effects for the repetition priming/repetition suppression phenomena in the context of task-switching.

## Conclusion

We used ERPs to demonstrate that repetition processes in task-switching are affected by the specific effects of long and short RSI and CSI combinations. Behavioral repetition priming occurred in the short CSI conditions, and ERP repetition suppression occurred in the LS condition (long RSI and short CSI). These findings suggest that repetition priming/repetition suppression phenomena do not always occur by repeated exposure to the same stimuli (or task), but rather depend on the extent to which the cue-stimulus transformation processes have been accomplished and whether continued activation from the previous trial has dissipated, as determined by the duration of CSI and RSI, respectively.

## Author Contributions

MW and LL conceived and designed the experiments. MW performed the experiments. MW and PY analyzed the data. Q-JZ, MW and ZJ wrote the main manuscript text. All authors reviewed the manuscript.

## Conflict of Interest Statement

The authors declare that the research was conducted in the absence of any commercial or financial relationships that could be construed as a potential conflict of interest.
